# 5,5-Bis(hydroxy­meth­yl)-3-methyl­cyclo­hex-2-enone

**DOI:** 10.1107/S1600536808025063

**Published:** 2008-08-13

**Authors:** Dongmei Cui, Qian Wang, Chen Zhang, Jianming Gu

**Affiliations:** a College of Pharmaceutical Science, Zhejiang University of Technology, Hangzhou 310014, People’s Republic of China; b Zhejiang University, Hangzhou 310058, People’s Republic of China

## Abstract

In the title compound, C_9_H_14_O_3_, the cyclo­hexenone ring has an envelope conformation; the flap atom (with the hydroxy­methyl groups attached) is displaced by 0.582 (4) Å from the plane of the other five ring atoms. The crystal structure contains an inter­molecular O—H⋯O hydrogen-bonded ring.

## Related literature

For related literature, see: Aghil *et al.* (1992 ▶); Hu *et al.* (2003 ▶); Li & Strobel (2001 ▶); Luu *et al.* (2004 ▶).
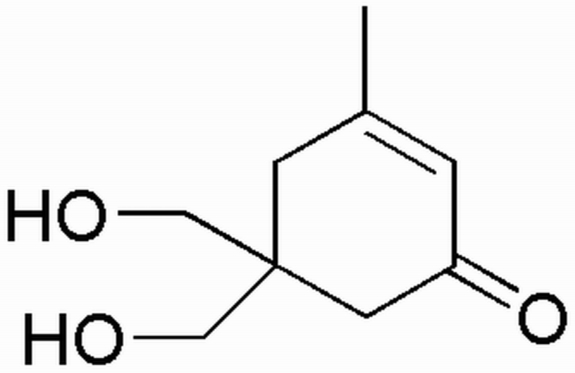


## Experimental

### 

#### Crystal data

C_9_H_14_O_3_
*M* *_r_* = 170.21
Triclinic, 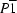

*a* = 5.9791 (3) Å
*b* = 6.2251 (1) Å
*c* = 13.7493 (8) Å
α = 90.8104 (17)°
β = 91.3285 (12)°
γ = 117.0728 (15)°
*V* = 455.38 (4) Å^3^
*Z* = 2
Mo *K*α radiation
μ = 0.09 mm^−1^
*T* = 296 (1) K
0.43 × 0.40 × 0.20 mm

#### Data collection

Rigaku R-AXIS RAPID diffractometer
Absorption correction: multi-scan (*ABSCOR*; Higashi, 1995 ▶) *T*_min_ = 0.958, *T*_max_ = 0.982
4514 measured reflections
2060 independent reflections
1432 reflections with *F* ^2^ > 2σ(*F* ^2^)
*R*_int_ = 0.018

#### Refinement

*R*[*F* ^2^ > 2σ(*F* ^2^)] = 0.054
*wR*(*F* ^2^) = 0.195
*S* = 1.01
2060 reflections
110 parameters
H-atom parameters constrained
Δρ_max_ = 0.29 e Å^−3^
Δρ_min_ = −0.23 e Å^−3^

### 

Data collection: *PROCESS-AUTO* (Rigaku, 1998 ▶); cell refinement: *PROCESS-AUTO*; data reduction: *CrystalStructure* (Rigaku/MSC, 2004 ▶) and Larson (1970 ▶); program(s) used to solve structure: *SIR97* (Altomare *et al.*, 1999 ▶); program(s) used to refine structure: *CRYSTALS* (Betteridge *et al.*, 2003 ▶); molecular graphics: *ORTEP-3 for Windows* (Farrugia, 1997 ▶); software used to prepare material for publication: *CrystalStructure*.

## Supplementary Material

Crystal structure: contains datablocks global, I. DOI: 10.1107/S1600536808025063/ez2128sup1.cif

Structure factors: contains datablocks I. DOI: 10.1107/S1600536808025063/ez2128Isup2.hkl

Additional supplementary materials:  crystallographic information; 3D view; checkCIF report

## Figures and Tables

**Table 1:** Hydrogen-bond geometry (Å, °)

*D*—H⋯*A*	*D*—H	H⋯*A*	*D*⋯*A*	*D*—H⋯*A*
O2—H201⋯O3^i^	0.92	1.85	2.738 (2)	163
O3—H301⋯O2^ii^	0.95	1.84	2.733 (2)	155

Symmetry codes: (i) 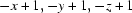; (ii) 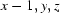.

**Fig. 1.**
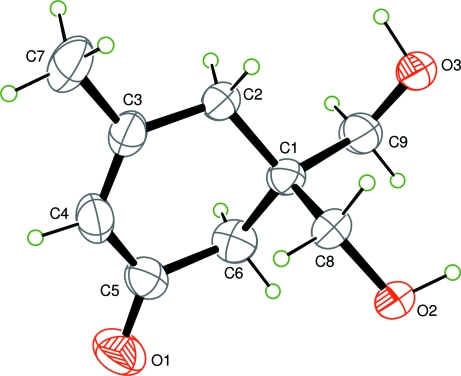
The unit of (I) with atom labels, showing 50% probability displacement ellipsoids.

**Fig. 2.**
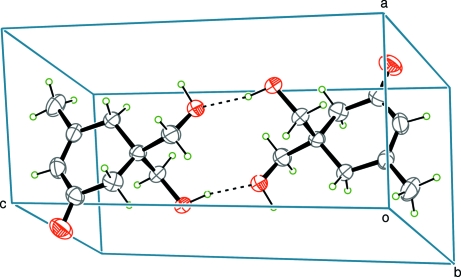
A partial packing diagram viewed along the b axis. Hydrogen bonds are drawn as dashed lines.

## supplementary crystallographic information

### Comment

Functionalized cyclohex-2-enone derivatives can be used as precursors in the syntheses of some complex compounds, such as vitamin E, amino acids, terpenes *etc*. (Hu *et al.*, 2003). In addition, cyclohex-2-enone derivatives have been shown to have a wide range of biological activities such as antimicrobial (Li *et al.*, 2001) and anticancer (Aghil *et al.*, 1992) activities, and are involved in the protection of cerebral neurocytes (Luu *et al.*, 2004). We are interested in their further pharmaceutical activity.

In this paper, we present an X-ray crystallographic analysis of the title compound (I) (Fig. 1). The cyclohexenone ring has an envelope conformation, such that the plane which is composed of atoms C1, C2 and C6 (forming the flap) and the C2, C3, C4, C5, C6 plane form a dihedral angle of 41.80 (4)°. Two molecules are linked together through O—H···O interactions. Since each molecule contains a hydrogen-bond donor group (–OH) at one end and an acceptor (–OH) at the other, a ring of four H-bonds is formed between these two molecules and a neighboring pair in the crystal lattice (Fig. 2).

### Experimental

A solution of 4,4-bis(hydroxymethyl)-2,6-heptanedione(188 mg, 1 mmol) and sodium methoxide (54 mg, 1 mmol) in methanol (10 ml) was heated at 323 K for 4 h. The reaction mixture was acidified with dilute aqueous HCl, then concentrated and partitioned between water and dichloromethane. The pure product was obtained through silica gel chromatography (eluant petroleum ether/ethyl acetate, 1:1), and diffraction quality crystals were obtained by slow evaporation of a dichloromethane / petroleum ether (1:3) solution at room temperature.

### Refinement

All H atoms were placed in calculated positions, with C—H distances in the range 0.93–0.98Å and included in the final cycles of refinement in the riding-model approximation, with *U*_iso_(H) = 1.2Ueq(C).

### Crystal data

**Table:**

C_9_H_14_O_3_	*Z* = 2
*M**_r_* = 170.21	*F*_000_ = 184.00
Triclinic, *P*1	*D*_x_ = 1.241 Mg m^−^^3^
Hall symbol: -P 1	Mo *K*α radiation λ = 0.71075 Å
*a* = 5.9791 (3) Å	Cell parameters from 3491 reflections
*b* = 6.22510 (10) Å	θ = 3.7–27.4º
*c* = 13.7493 (8) Å	µ = 0.09 mm^−^^1^
α = 90.8104 (17)º	*T* = 296 (1) K
β = 91.3285 (12)º	Chunk, colorless
γ = 117.0728 (15)º	0.43 × 0.40 × 0.20 mm
*V* = 455.38 (4) Å^3^	

### Data collection

**Table:**

Rigaku R-AXIS RAPID diffractometer	1432 reflections with *F*^2^ > 2σ(*F*^2^)
Detector resolution: 10.00 pixels mm^-1^	*R*_int_ = 0.018
ω scans	θ_max_ = 27.5º
Absorption correction: multi-scan(ABSCOR; Higashi, 1995)	*h* = −7→7
*T*_min_ = 0.958, *T*_max_ = 0.982	*k* = −8→8
4514 measured reflections	*l* = −17→17
2060 independent reflections	

### Refinement

**Table:**

Refinement on *F*^2^	*w* = 1/[0.0027*F*_o_^2^ + 5σ(*F*_o_^2^) + 1]/(4*F*_o_^2^)
*R*[*F*^2^ > 2σ(*F*^2^)] = 0.054	(Δ/σ)_max_ < 0.001
*wR*(*F*^2^) = 0.195	Δρ_max_ = 0.29 e Å^−^^3^
*S* = 1.01	Δρ_min_ = −0.23 e Å^−^^3^
2060 reflections	Extinction correction: Larson (1970)
110 parameters	Extinction coefficient: 107 (30)
H-atom parameters constrained	

### Special details

**Table:**

Refinement. Refinement using all reflections. The weighted *R*-factor (*wR*) and goodness of fit (*S*) are based on *F*^2^. *R*-factor (gt) are based on *F*. The threshold expression of *F*^2^ > 2.0 σ(*F*^2^) is used only for calculating *R*-factor (gt).

### Fractional atomic coordinates and isotropic or equivalent isotropic displacement parameters (Å^2^)

**Table:**

	*x*	*y*	*z*	*U*_iso_*/*U*_eq_	
O1	1.1756 (3)	1.1069 (3)	0.16817 (14)	0.0617 (6)	
O2	0.8610 (2)	0.5583 (3)	0.38179 (12)	0.0518 (5)	
O3	0.3183 (3)	0.5928 (4)	0.43726 (12)	0.0625 (6)	
C1	0.6300 (3)	0.7646 (4)	0.31049 (14)	0.0342 (5)	
C2	0.4284 (4)	0.7285 (4)	0.23242 (14)	0.0382 (6)	
C3	0.5141 (4)	0.7644 (4)	0.13114 (16)	0.0395 (6)	
C4	0.7599 (4)	0.8911 (4)	0.11129 (17)	0.0459 (6)	
C5	0.9546 (4)	1.0036 (4)	0.18614 (18)	0.0418 (6)	
C6	0.8686 (4)	0.9901 (4)	0.29092 (17)	0.0435 (6)	
C7	0.3125 (5)	0.6558 (5)	0.05324 (18)	0.0575 (8)	
C8	0.6772 (4)	0.5422 (4)	0.31014 (16)	0.0391 (6)	
C9	0.5370 (4)	0.7935 (5)	0.41112 (17)	0.0497 (7)	
H4	0.8057	0.9066	0.0466	0.055*	
H21	0.2949	0.5649	0.2365	0.046*	
H22	0.3628	0.8418	0.2467	0.046*	
H61	1.0013	0.9953	0.3343	0.052*	
H62	0.8404	1.1291	0.3044	0.052*	
H71	0.3875	0.6827	−0.0093	0.069*	
H72	0.2024	0.7294	0.0564	0.069*	
H73	0.2183	0.4855	0.0626	0.069*	
H81	0.5204	0.4012	0.3225	0.047*	
H82	0.7344	0.5243	0.2465	0.047*	
H91	0.6684	0.8209	0.4596	0.060*	
H92	0.5042	0.9324	0.4103	0.060*	
H201	0.7736	0.4867	0.4357	0.067*	
H301	0.1888	0.6149	0.4049	0.081*	

### Atomic displacement parameters (Å^2^)

**Table:**

	*U*^11^	*U*^22^	*U*^33^	*U*^12^	*U*^13^	*U*^23^
O1	0.0437 (10)	0.0582 (12)	0.0750 (14)	0.0149 (8)	0.0195 (9)	0.0154 (10)
O2	0.0403 (9)	0.0843 (13)	0.0433 (9)	0.0383 (9)	0.0072 (7)	0.0232 (8)
O3	0.0444 (9)	0.1166 (18)	0.0398 (9)	0.0471 (11)	0.0123 (7)	0.0284 (10)
C1	0.0325 (10)	0.0441 (13)	0.0296 (10)	0.0205 (9)	0.0006 (8)	−0.0006 (8)
C2	0.0357 (10)	0.0514 (14)	0.0324 (11)	0.0239 (10)	0.0002 (8)	0.0044 (9)
C3	0.0480 (12)	0.0438 (13)	0.0328 (11)	0.0263 (11)	−0.0014 (9)	0.0041 (9)
C4	0.0556 (14)	0.0524 (15)	0.0333 (11)	0.0272 (12)	0.0097 (10)	0.0090 (10)
C5	0.0422 (12)	0.0347 (12)	0.0508 (13)	0.0190 (10)	0.0108 (10)	0.0086 (10)
C6	0.0423 (12)	0.0411 (13)	0.0437 (13)	0.0163 (10)	−0.0014 (10)	−0.0049 (10)
C7	0.0663 (17)	0.0717 (19)	0.0387 (13)	0.0360 (15)	−0.0145 (12)	−0.0003 (12)
C8	0.0371 (11)	0.0488 (14)	0.0366 (11)	0.0238 (10)	−0.0010 (9)	0.0071 (9)
C9	0.0473 (13)	0.0781 (19)	0.0333 (12)	0.0367 (13)	0.0062 (10)	0.0024 (12)

### Geometric parameters (Å, °)

**Table:**

O1—C5	1.211 (2)	O3—H301	0.948
O2—C8	1.426 (3)	C2—H21	0.970
O3—C9	1.395 (2)	C2—H22	0.970
C1—C2	1.530 (3)	C4—H4	0.930
C1—C6	1.512 (2)	C6—H61	0.970
C1—C8	1.534 (4)	C6—H62	0.970
C1—C9	1.541 (3)	C7—H71	0.960
C2—C3	1.480 (3)	C7—H72	0.960
C3—C4	1.351 (3)	C7—H73	0.960
C3—C7	1.495 (3)	C8—H81	0.970
C4—C5	1.445 (3)	C8—H82	0.970
C5—C6	1.531 (3)	C9—H91	0.970
O2—H201	0.915	C9—H92	0.970
			
C2—C1—C6	109.76 (18)	C3—C4—H4	118.6
C2—C1—C8	109.12 (18)	C5—C4—H4	118.6
C2—C1—C9	109.4 (2)	C1—C6—H61	108.4
C6—C1—C8	110.8 (2)	C1—C6—H62	108.4
C6—C1—C9	108.79 (18)	C5—C6—H61	108.4
C8—C1—C9	109.0 (2)	C5—C6—H62	108.4
C1—C2—C3	115.5 (2)	H61—C6—H62	109.5
C2—C3—C4	121.45 (19)	C3—C7—H71	109.5
C2—C3—C7	115.92 (19)	C3—C7—H72	109.5
C4—C3—C7	122.6 (2)	C3—C7—H73	109.5
C3—C4—C5	122.9 (2)	H71—C7—H72	109.5
O1—C5—C4	122.5 (2)	H71—C7—H73	109.5
O1—C5—C6	120.8 (2)	H72—C7—H73	109.5
C4—C5—C6	116.7 (2)	O2—C8—H81	108.6
C1—C6—C5	113.84 (17)	O2—C8—H82	108.6
O2—C8—C1	113.12 (18)	C1—C8—H81	108.6
O3—C9—C1	113.6 (2)	C1—C8—H82	108.6
C8—O2—H201	105.8	H81—C8—H82	109.5
C9—O3—H301	103.4	O3—C9—H91	108.4
C1—C2—H21	107.9	O3—C9—H92	108.4
C1—C2—H22	107.9	C1—C9—H91	108.4
C3—C2—H21	107.9	C1—C9—H92	108.4
C3—C2—H22	107.9	H91—C9—H92	109.5
H21—C2—H22	109.5		
			
C2—C1—C6—C5	−50.0 (3)	C8—C1—C9—O3	−58.2 (2)
C6—C1—C2—C3	44.7 (3)	C9—C1—C8—O2	−59.9 (2)
C2—C1—C8—O2	−179.24 (16)	C1—C2—C3—C4	−19.9 (4)
C8—C1—C2—C3	−76.9 (2)	C1—C2—C3—C7	161.0 (2)
C2—C1—C9—O3	61.0 (3)	C2—C3—C4—C5	−1.2 (4)
C9—C1—C2—C3	164.0 (2)	C7—C3—C4—C5	177.8 (3)
C6—C1—C8—O2	59.8 (2)	C3—C4—C5—O1	176.3 (3)
C8—C1—C6—C5	70.6 (2)	C3—C4—C5—C6	−4.6 (4)
C6—C1—C9—O3	−179.1 (2)	O1—C5—C6—C1	−149.4 (2)
C9—C1—C6—C5	−169.7 (2)	C4—C5—C6—C1	31.5 (3)

### Hydrogen-bond geometry (Å, °)

**Table:**

*D*—H···*A*	*D*—H	H···*A*	*D*···*A*	*D*—H···*A*
O2—H201···O3^i^	0.92	1.85	2.738 (2)	163
O3—H301···O2^ii^	0.95	1.85	2.733 (2)	155

Symmetry codes: (i) −*x*+1, −*y*+1, −*z*+1; (ii) *x*−1, *y*, *z*.
